# Ferumoxytol-enhanced three-dimensional magnetic resonance imaging of carotid atheroma- a feasibility and temporal dependence study

**DOI:** 10.1038/s41598-020-58708-x

**Published:** 2020-02-04

**Authors:** Ammara Usman, Andrew J. Patterson, Jianmin Yuan, Alison Cluroe, Ilse Patterson, Martin J. Graves, Jonathan H. Gillard, Umar Sadat

**Affiliations:** 1 University Department of Radiology, Addenbrooke’s Hospital, Cambridge, CB2 0QQ UK; 2 Department of Pathology, Addenbrooke’s Hospital, Cambridge, CB2 0QQ UK; 3 Christ’s College, Cambridge, CB2 3BU UK; 4 University Department of Surgery, Addenbrooke’s Hospital, Cambridge, CB2 0QQ UK

**Keywords:** Diagnostic markers, Diagnostic markers

## Abstract

Ferumoxytol is an ultrasmall super paramagnetic particles of iron oxide (USPIO) agent recently used for magnetic resonance (MR) vascular imaging. Other USPIOs have been previously used for assessing inflammation within atheroma. We aim to assess feasibility of ferumoxytol in imaging carotid atheroma (with histological assessment); and the optimum MR imaging time to detect maximum quantitative signal change post-ferumoxytol infusion. Ten patients with carotid artery disease underwent high-resolution MR imaging of their carotid arteries on a 1.5 T MR system. MR imaging was performed before and at 24, 48, 72 and 96 hrs post ferumoxytol infusion. Optimal ferumoxytol uptake time was evaluated by quantitative relaxometry maps indicating the difference in T_2_* (ΔT_2_*) and T_2_ (ΔT_2_) between baseline and post-Ferumoxytol MR imaging using 3D DANTE MEFGRE qT_2_*w and iMSDE black-blood qT_2_w sequences respectively. 20 patients in total (10 symptomatic and 10 with asymptomatic carotid artery disease) had ferumoxytol-enhanced MR imaging at the optimal imaging window. 69 carotid MR imaging studies were completed. Ferumoxytol uptake (determined by a decrease in ΔT_2_* and ΔT_2_) was identified in all carotid plaques (symptomatic and asymptomatic). Maximum quantitative decrease in ΔT_2_* (10.4 [3.5–16.2] ms, p < 0.001) and ΔT_2_ (13.4 [6._2_–18.9] ms; p = 0.001) was found on carotid MR imaging at 48 hrs following the ferumoxytol infusion. Ferumoxytol uptake by carotid plaques was assessed by histopathological analysis of excised atheroma. Ferumoxytol**-**enhanced MR imaging using quantitative 3D MR pulse sequences allows assessment of inflammation within carotid atheroma in symptomatic and asymptomatic patients. The optimum MR imaging time for carotid atheroma is 48 hrs after its administration.

## Introduction

Immune-mediated inflammation^1^ and related neovascularization^2^ play crucial role in the progression of atherosclerotic disease processes^3^. Macrophages are the major inflammatory mediators of this process^4^ which become concentrated at the plaque shoulder and necrotic lipid core that makes the plaque more vulnerable to rupture and thromboembolic sequelae^5^.

Magnetic resonance (MR) imaging using targeted contrast medium such as ultrasmall superparamagnetic particles of iron oxide (USPIOs) have demonstrated promising results in investigating the pathophysiology of atherosclerosis^6,7^ and in the assessment of the effectiveness of anti-atherosclerotic treatments^8^. The physiochemical properties of USPIOs attribute to their effective uptake by macrophages and their longer plasma half-life makes them suitable for atheroma imaging. The superparamagnetic core of USPIOs alters the magnetic susceptibility by creating an imbalance of the externally applied magnetic field, which in turn leads to signal reduction on T_2_ and T_2_*-weighted MR images. The areas containing these particles display rapid transverse relaxation and present as hypointense signal changes (i.e. negative contrast) on T_2_ and T_2_* weighted imaging and reduction in quantitative T_2_ and T_2_^*^ relaxation times.

Several MR imaging studies have demonstrated the optimal time window for detection of macrophages following the infusion of ferumoxtran-10 in patients with carotid atherosclerotic disease^9,10^. USPIO-enhanced MR imaging has also effectively demonstrated the systemic inflammatory nature of atherosclerosis affecting various arterial beds simultaneously^6^. Using serial USPIO-enhanced MR imaging over a 3-month period in symptomatic patients, a significant reduction in carotid plaque inflammation with high-dose statin-lowering therapy compared with low-dose therapy had also been reported^8^. Despite, having potential benefit for imaging atherosclerotic tissue and having an acceptable safety profile, Ferumoxtran-10 is no longer available.

Ferumoxytol (AMAG Pharmaceuticals, Lexington, MA, USA) is a USPIO that has obtained approval in the treatment of iron deficiency anaemia in patients with chronic renal failure. Ferumoxytol holds promise as an MR CM, however, it differs from Ferumoxtran-10 in various physicochemical properties. The plasma half -life of Ferumoxytol is (10–14 hrs) compared to (≈ 24 hrs) of Ferumoxtran-10 and it has different relaxivity (r_1_ = 15 mM^−1^s^−1^, r_2_ = 89 mM^−1^s^−1^) and r_1_ = 9.9 mM^−1^s^−1^, r_2_ = 65 mM^−1^s^−1^ respectively)^11^. Based on these differences, it can be hypothesised that ferumoxytol has a different optimal post-infusion imaging window.

Previously, there have been reports of the use of ferumoxytol in assessing arterial wall inflammation in carotid arteries^12^ and in aorta^13^. These studies however did not assess temporal dependence of ferumoxytol i.e. optimal imaging time post administration. Semi quantitative MR pulse sequences were used which also have limitations as discussed below. In the absence of the key temporal dependence information of ferumoxytol (aorta and/or carotid), it has been quiet premature to conduct any large scale study^14^, making the methodology of the study flawed and results unreliable.

In this study we aim to:

1. Determine whether ferumoxytol can be used for MR imaging of carotid plaques.
2. Assess the optimum MR imaging time to detect maximum signal change post ferumoxytol administration, using 3D qT_2_ and qT_2_* imaging.
3. To assess the ferumoxytol enhanced-MR imaging quantified signal drop (representative of underlying plaque inflammation) within carotid atheroma in the patients with symptomatic and asymptomatic carotid artery disease.

## Methods

Ten consecutive patients (8 males and 2 females) with moderate to severe duplex-ultrasound confirmed carotid artery disease (i.e. 50–99%) were recruited in the first part of study. Each patient underwent a baseline pre-ferumoxytol MR imaging; and post-ferumoxytol MR imaging was performed at 24, 48, 72 and 96 hrs following infusion for assessing feasibility of this imaging method and to calculate the temporal dependence.

In the second part of this exploratory study, 10 symptomatic and 10 asymptomatic patients with moderate to severe Duplex US confirmed carotid artery disease (i.e. 50–99%) had been recruited in total. Recruitment was consecutive. Among these 20, 10 had already been participants of the initial temporal dependence study. Each patient underwent a baseline pre-ferumoxytol MR imaging; and post-ferumoxytol MR imaging was performed at the optimal MR imaging time as determined by the temporal dependence investigation

This study was approved by the central Cambridge research ethics research committee. All methods were performed in accordance with the relevant guidelines and regulations. All study participants provided written informed consent.

A >50% internal carotid artery stenosis by duplex ultrasound [based on North American Symptomatic Carotid Endarterectomy Trial criteria] and ability to sign informed consent were necessary requirements for inclusion. Exclusion criteria were (i) history of atopy, asthma or allergic reaction to contrast media, iron or dextran, (ii) known and documented history of haemochromatosis (iii) patients with immune or inflammatory conditions e.g. systemic lupus erythematous, rheumatoid arthritis (iv) standard MR exclusion criteria.

### MR imaging

Imaging was performed on a 1.5 Tesla whole body MR imaging system (MR450w, GE Healthcare, Waukesha, WI), using a 4-channel phased-array neck coil (PACC, Machnet, Roden, The Netherlands). Movement artefact was minimized using a dedicated vacuum-based head restraint system (VAC-LOK Cushion; Oncology Systems Ltd, Shrewsbury, United Kingdom) which serves to maintain the head and neck in a comfortable position and fix the coil position.

Localiser and 2D time-of-flight sequences were performed to locate the carotid bifurcations. The following pulse sequences were used: T_1_w images were acquired using a 3D variable refocusing flip angle fast spin echo sequence with Delayed Alternating with Nutation for Tailored Excitation blood suppression preparation. qT2* mapping was performed using a multi-echo fast gradient echo sequence using DANTE blood suppression preparation^15^. Six echoes were used as in our experience it is a good balance for the required imaging field of view and matrix size for the carotid study.

Finally, quantitative T2 (qT2) was performed with a multi-echo variable refocussing flip angle FSE sequence with improved Motion-Sensitized Driven-Equilibrium. The accuracy and repeatability of improved Motion-Sensitized Driven-Equilibrium prepared 3D variable refocussing flip angle fast spin echo for T2 mapping has previously been reported^16^. We used three echo times to generate qT2 maps after practical consideration of scanning time and accuracy. We believe that this sequence is the limit in terms of scan time and patient tolerance. This sequence is 3D with high spatial resolution which allows good determination of vessel wall plaque components while keeping the scan time short as short as possible to minimize neck motion.

The detailed parameters of the pulse sequences used are tabulated in the Table 1.

**Table 1:** Summary of imaging parameters for the multi-contrast magnetic resonance (MR) imaging protocol at 1.5 T.

Contrast weighting	T_1_w	qT2*	qT2
Sequence	3D Variable Refocusing Flip Angle FSE	3D multi-echo fast gradient echo	3D Variable Refocusing Flip Angle FSE
Acquisition plane	Axial	Axial	Axial
Blood suppression method	delays alternating with nutation for tailored excitation	delays alternating with nutation for tailored excitation	improved motion sensitive driven equilibrium
Echoes per repetition time / echo train length	24	6	40
Echo time(s) (ms)	15.1	4.9,10.4,15.9,21.3,26.8,32.2	26.3, 56.3, 86.3
Repetition time (ms)	580	58.1	2000
Flip angle	variable	15	variable
Acquisition matrix	224 × 224	224 × 224	224 × 224
Acquisition time	5 min 47 s	7 min 52 s	8 min 55 s
Field of view (cm)	14 × 14	14 × 14	14 × 14
Pixel size (mm)	0.625 × 0.625	0.625 × 0.625	0.625 × 0.625
Slice Thickness (mm)	1.4	2.0	2.0
Number of excitations	2	1	1

### Ferumoxytol administration

Patient’s vital signs (i.e. pulse rate, blood pressure and respiratory rate) were recorded at three-time intervals (within 15 minutes) before the ferumoxytol infusion. The USPIO agent, Ferumoxytol (AMAG, Canada) was obtained as a liquid preparation in 17 ml vials. The agent was further diluted in 250 ml of normal saline and administered as a slow infusion through an indwelling large bore intravenous cannula over 30 minutes. The dose administered was 5 mg/kg. Vitals were monitored every 10 minutes during the infusion and up to 30 min after the ferumoxytol administration. The physiochemical properties and safety data for Ferumoxytol has been published previously^11,17,18^.

### Image analysis

Initial image analysis was performed by the first author (AU) and cross-checked by two experienced MR readers (JHG, US) with greater than 30 yrs combined experience between them. Image quality was rated per vessel for each contrast weighting on a 5-point scale (1 = poor, 5 = excellent) dependant on the overall signal to noise ratio, clarity of the lumen and the vessel wall boundaries. Images with an image quality of ≤ 2 were excluded from the study.

Pre- and post-USPIO images were manually co-registered according to plaque morphology and distance from the carotid bifurcation at the time of imaging. Slice position relative to the carotid bifurcation were assigned ordinal numbers to enable pre- and post-infusion slice matching. Images were segmented on the first echo of the qT2 and qT2* sequences. Following the identification of the carotid plaque region on T2 and T2* weighted images, the lumen and vessel wall contours were manually drawn using a closed polygon to define the arterial wall ROI using OsiriX (OsiriX 5.5.2, Pixmeo, Geneva, Switzerland). Region of interest (ROI) was defined as a subset of the acquired MR image or a dataset used for identification of carotid plaque region based on morphology and anatomical landmarks.

A region was also drawn in an artefact-free background by the same observer, the standard deviation of this background region was used to quantify noise (defined as σ). A region was also drawn in the sternocleidomastoid muscle and the mean signal intensity was reported S_wall_. The SNR was calculated using a four-channel phased array correction factor: SNR_wall_ = 0.659 × S_wall_/σ^19^.

Digital Imaging and Communications in Medicine images were imported into an in-house software developed in Matlab (The Math Works, Version R2013b, Natick, MA, USA) to perform T2*and T2 quantification. ROI files were exported from OsiriX and were imported into in-house Matlab software to generate quantitative relaxometry maps (T2*/R2*, T2/R2) and to calculate the mean T2* and T2 values.

### Histological analysis

Carotid plaques of patients scheduled for carotid endarterectomy were retrieved and processed according to the protocol designed by the consultant histopathologist (AC) to assess for the presence of ferumoxytol within atheroma. The specimen was placed in formalin and following overnight fixation place in Ethylenediaminetetraacetic acid for approximately 24 hrs for decalcification if necessary. Subsequently, 3 mm transverse sections were cut through the specimen and multiple slices were processed in a cassette. These processed specimens were embedded in paraffin blocks. From these blocks further 4 sections were cut at 3.5 micrometres. These sections were dehydrated and underwent haematoxylin and eosin (H&E), Perls reagent, elastin van Gieson (EVG) stains and immunostaining for macrophages (CD 68 marker). Histological sections were reviewed by the consultant histopathologist (AC) using a Nikon Eclipse 80i microscope at ×10, ×25 and ×40. Ferumoxytol accumulation was confirmed by Perls’ positive staining and histological sections were classified as positive or negative accordingly.

### Statistical analysis

Ferumoxytol uptake was quantified by the absolute change in T_2_* (ΔT_2_*) and T_2_ (ΔT_2_) between the pre and post-ferumoxytol MR images measured for each arterial wall segment (ROI) with a decrease in T_2_* and T_2_ values indicating the ferumoxytol uptake. The analysis was quantified twice: whole plaque measurements were derived from all ROIs from slices contain plaque with sufficient image quality, and this process was repeated using only slice matched ROIs across the multiple visits. Continuous variables are presented as median (interquartile range-IQR). Data normality was assessed by Shapiro–Wilk’s test. Paired Wilcoxon Signed Rank Test was used to compare the changes in T_2_ and T_2_* values relative to baseline examination at each follow-up visit. Spearman’s correlation was used to evaluate the correlation in ferumoxytol uptake at different time frames on both T_2_ and T_2_* images.

For the comparison between symptomatic and asymptomatic cohort of patients, analyses was performed as above, to obtain whole plaque measurements for the two visits. A paired t-test was used to compare the changes in T_2_ and T_2_* values relative to baseline and post contrast imaging session and unpaired t-test to compare the difference between the groups. P values <0.05 were defined as statistically significant. The statistical analysis was performed in R (version 3.2.1).

## Results

### Quantitative MR image analysis- feasibility and temporal dependence assessment

Nine of the ten patients completed all of the MR examinations. One patient was unable to attend the final 96 hrs imaging session. The patient demographics and co-morbidities are presented in Table 2. The scheduled 24, 48, 72 and 96 post-infusion scans occurred at 24.6 ± 2.1, 48.5 ± 1.5, 73.7 ± 1.6 and 98.4 ± 1.7 hrs post-infusion.

**Table 2:** Patient demographics and comorbidities.

Characteristics	n (%)
Number of patients	10
Male/Female	8/2
Median age (years) (interquartile range)	73 (67–75)
Mean luminal stenosis (North American Symptomatic Carotid Endarterectomy Trial) (%) [IQR]	56.9 (50.2–67.4)
TIA/non-disabling stroke	10 (100%)
Hyperlipidaemia	6 (60%)
Hypertension	7 (70%)
Diabetes Mellitus	2 (20%)
Peripheral arterial disease	2 (20%)
Previous coronary revascularisation	1 (10%)
Ischaemic heart disease	3 (30%)
Atrial fibrillation	0
Statin use	10 (100%)
Clopidogrel	8 (80%)
Aspirin	6 (60%)
Current or ex-smoker	6 (60%)

In total 69 carotid MR imaging studies were completed, and MR images used for analyses.

**Figure 1:**
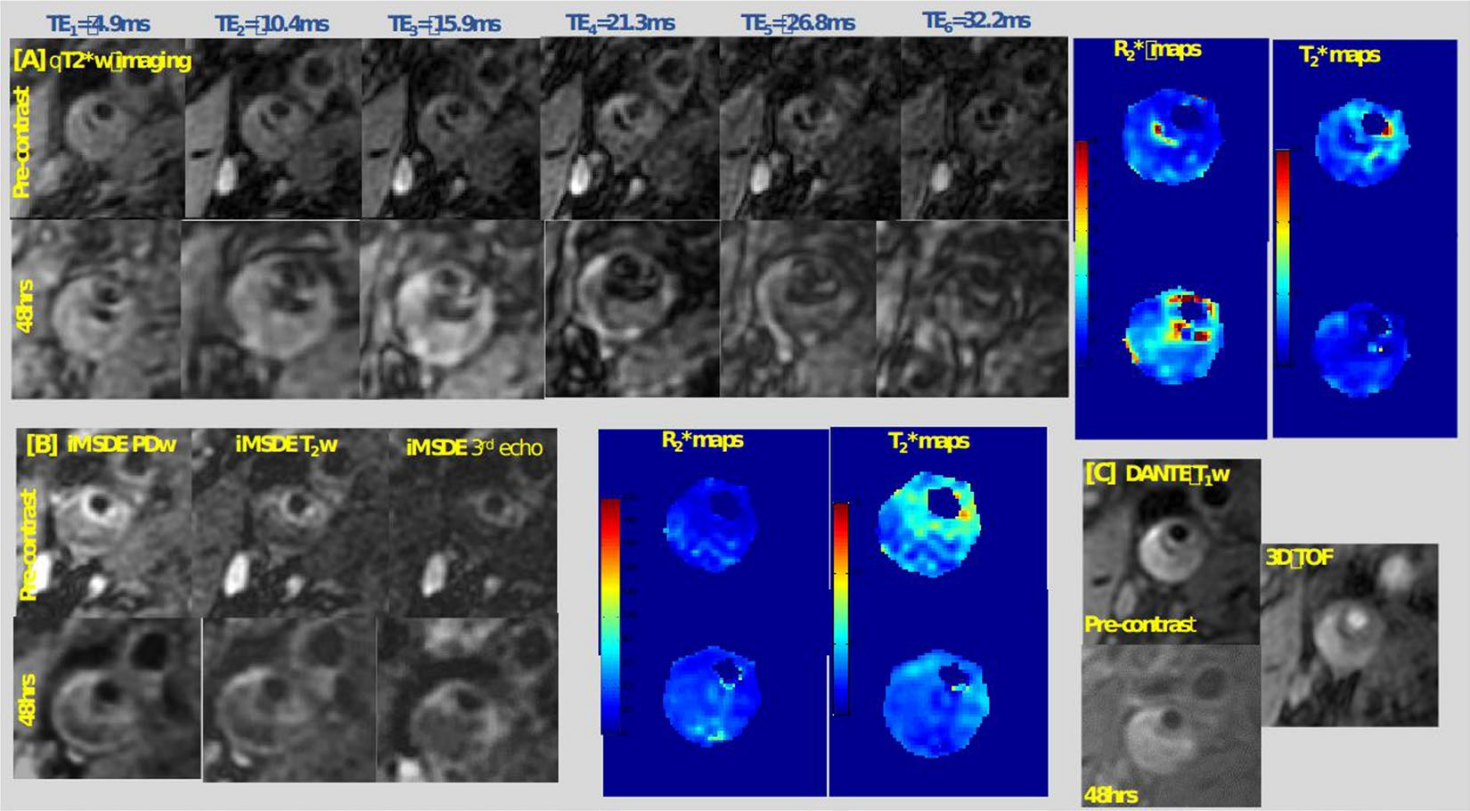
Detailed multi-contrast ferumoxytol-enhanced magnetic resonance imaging protocol of carotid atheroma demonstrating the qT_2_* with six echo times, qT_2_ with three echo times and T_1_ pulse sequence at baseline and 48hrs post ferumoxytol administration with corresponding R_2_* and T_2_*maps. This figure illustrates the detailed MR imaging protocol at baseline and 48 hrs after the administration of ferumoxytol in a patient with right internal carotid artery stenosis >70% as calculated by NASCET criteria. Panel [A] shows the multi-echo T_2_*/R_2_* mapping sequence with six echo times and the corresponding R_2_* and T_2_* maps before and at 48 hrs following the administration of ferumoxytol. Panel [B] shows the iMSDE PD and T_2_ mapping sequence and the corresponding R_2_ and T_2_ maps before and at 48 hrs following the ferumoxytol administration. Panel [C] demonstrates the 3D TOF (time of flight) and T_1_ images pre-contrast and at 48 hrs of ferumoxytol administration.

Among 10 patients, 18 plaques were identified (10 right-side and 8 left-side). Signal drop was identified in all plaques on qT_2_ and qT_2_* sequences as shown in Fig. 1.

**Figure 2:**
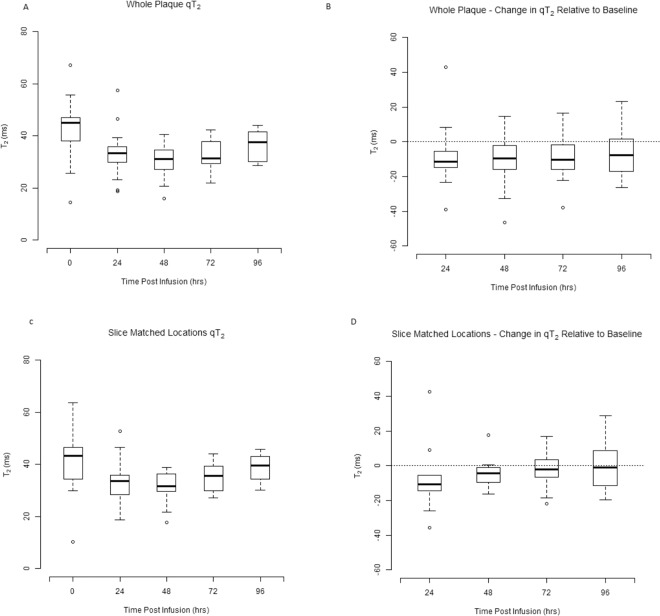
Box and whisker plots of whole plaque and slice matched quantitative T2 and change in quantitative T2 at 24, 48, 72 and 96 hrs post ferumoxytol administration relative to baseline. Figure illustrates the qT2 of (**A**) whole plaque (**C**) slice matched location with carotid bifurcation taken as a reference for slice match representing all 10 patients at baseline, 24, 48, 72 and 96 hrs post ferumoxytol administration. (**B**) Demonstrates the change in whole plaque qT2 at 24, 48, 72 and 96hrs relative to the baseline (p = 0.006), (p < 0.001), (p = 0.002) (p = 0.070) respectively (**D**) demonstrates the slice matched location change in qT2 at 24, 48, 72 and 96 hrs relative to the baseline (p = 0.053), (p = 0.015), (p = 0.093), (p = 0.770) respectively.

**Figure 3:**
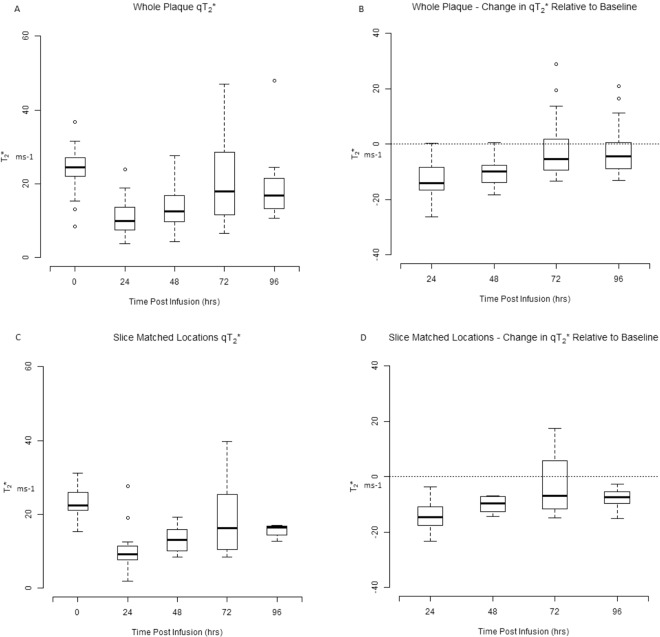
Box and whisker plots of whole carotid plaque and slice matched quantitative T2* and change in quantitative T2* at 24, 48, 72 and 96 hrs post ferumoxytol administration relative to baseline. Box and whisker plots illustrate the qT2* of (**A**) whole plaque (**C**) slice matched location with carotid bifurcation taken as a reference for slice match representing all 10 patients at baseline, 24, 48, 72 and 96 hrs post ferumoxytol administration. (**B**) Demonstrates the change in whole plaque qT2* at 24, 48, 72 and 96hrs relative to the baseline (p < 0.001), (p < 0.001), (p = 0.154) (p = 0.091) respectively (**D**) demonstrates the slice matched location change in qT2* at 24, 48, 72 and 96 hrs relative to the baseline (p = 0.001), (p < 0.001), (p = 0.222), (p = 0.020) respectively.

Mean T_2_ and T_2_* values of the whole plaque and slice matched location (see Figs. 2 and 3) on subsequent time points relative to baseline were used for statistical analysis. A decrease in qT_2_ and qT_2_* values was observed between 24–72 hrs with a positive correlation between the qT_2_ and qT_2_* values at 48 hrs post ferumoxytol infusion (Spearman’s r = 0.492, p = 0.039). A significant decrease in qT_2_ [13.4 [6.2 to 18.9] ms, (p = 0.001)] and qT_2_* [10.4 [3.5 to 16.2] ms, (p < 0.001)] was observed at 48 hrs post ferumoxytol administration.

**Figure 4:**
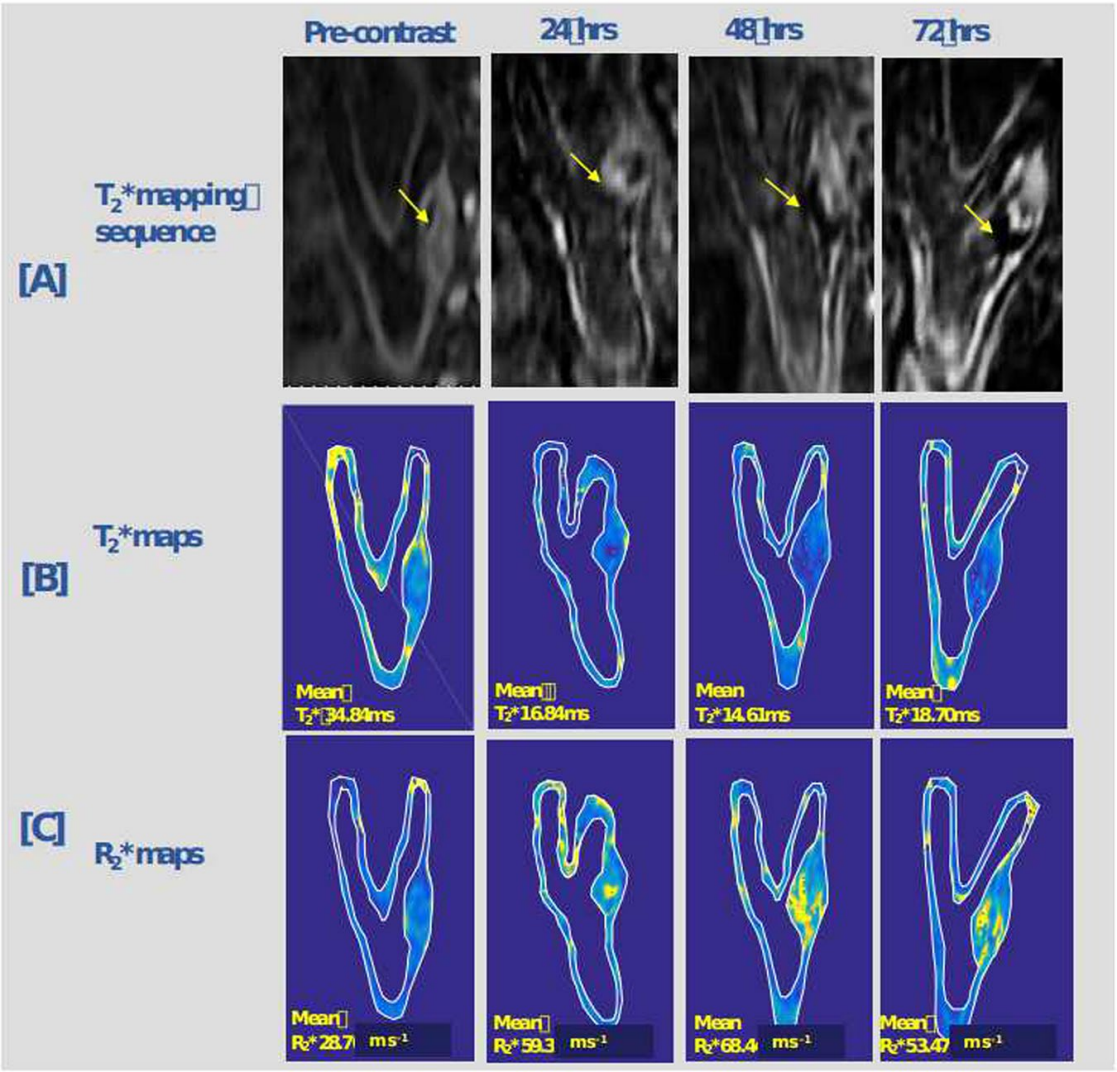
Oblique view of quantitative T2*mapping sequence of the carotid artery pre-contrast and 24, 48, 72 hrs post ferumoxytol with its corresponding T2* and R2* maps. An internal carotid artery plaque occupying more than 70% of the lumen is evident. [**A**] Demonstrates images acquired by the T2*/R2*mapping sequence at baseline and 24, 48 and 72 hrs following ferumoxytol infusion. Note the signal void at 48hrs-72hrs (yellow arrow) reflecting ferumoxytol uptake, [**B**] shows the corresponding oblique T2* maps with relative decrease in T2* values and maximum decrease at 48 hrs, [**C**] demonstrates the corresponding increase in R2*values with maximum increase at 48 hrs. The maximum T2* drop was seen on 48 hrs which also corresponds to the maximum R2* increase at the same time frame.

**Figure 5:**
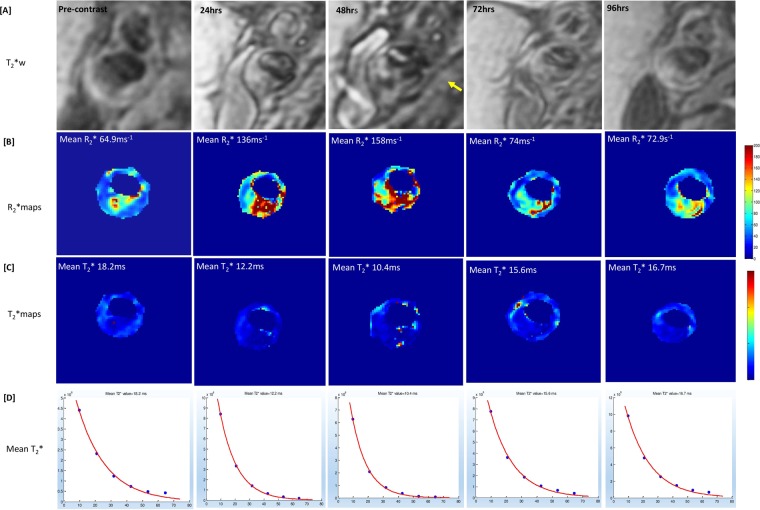
Ferumoxytol-enhanced magnetic resonance images of carotid atheroma with corresponding T2*/R2* maps and T2* graphs at baseline and 24, 48, 72 and 96 hrs of ferumoxytol administration demonstrating the maximum R2* increase of the atherosclerotic plaque after ferumoxytol administration at 48 hrs. [**A**] Images of the multi-echo T2*/R2* MR sequence at all 5 time points with signal void in carotid atheroma (yellow arrow) visible at 48 hrs following ferumoxytol administration. [**B**] Shows corresponding R2* maps (mean R* values) pre-contrast and at 24 hr, 48 hr, 72 hr and 96 hr following ferumoxytol administration. Note the maximum R2* increase of the atherosclerotic plaque after ferumoxytol administration at 48hrs, reflecting USPIO uptake. [**C**,**D**] Shows the corresponding T2* maps and graphs demonstrating maximum decrease in T2* values at 48 hr.

Using advanced 3D acquisition techniques, pixel-wise ΔT_2_/R_2_ and ΔT_2_*/R_2_* maps (R_2_* is the inverse of the mean T_2_*) from the pre and post contrast studies were generated (see Figs. 4 and 5). The median number of pixels per cross-sectional carotid wall area (mm^2^) was 1762 (Range: 657–3541). The minimum number of pixels per cross-sectional carotid wall area (mm2) was 675 which reduces the possibility of partial volume effect errors considering the small vessel size.

The maximum change in qT2 and qT2* relaxation is demonstrated in Tables 3 and 4 respectively.

**Table 3:** Quantitative T_2_ (qT_2_) changes relative to baseline examination at each follow-up visit. *Paired Wilcoxon Signed Rank Test, median [inter-quartiles].

MR Imaging	qT_2_ (ms)	ΔqT_2_ (ms)	p-value
Baseline	45.8 [39.8 to 56.5]	—	—
24hr-post	31.6 [28.7 to 36.2]	13.7 [3.3 to 28.2]	0.011*
48hrs-post	31.1 [29.0 to 35.7]	13.4 [6.2 to 18.9]	0.001*
72hrs-post	31.7 [28.8 to 38.2]	12.6 [2.6 to 22.2]	0.005*
96 hrs-post	34.9 [33.7 to 42.5]	10.5 [1.9 to 17.8]	0.015*

**Table 4:** Quantitative T_2_* (qT_2_*) changes relative to baseline examination at each follow-up visit. *Paired Wilcoxon Signed Rank Test, median [inter-quartiles].

MR Imaging	qT_2_* (ms)	ΔqT_2_* (ms)	p-value
Baseline	23.7 [19.9 to 25.4]	—	—
24hr-post	9.0 [6.9 to 13.6]	12.9 [9.5 to 16.4]	<0.001*
48hrs-post	12.4 [8.3 to 15.7]	10.4 [3.5 to 16.2]	<0.001*
72hrs-post	16.3 [10.6 to 28.0]	0.2 [-8.2 to 5.2]	0.832*
96hrs-post	12.9 [11.3 to 17.0]	8.7 [-0.5 to 12.2]	0.053*

As observed in Table 4, at 72 hrs post ferumoxytol unexpectedly small qT_2_* values were observed which was not in the case in qT_2_ values at the same time point (Table 3). Being an MR developmental study using a new CM, these unexpected results warrant further investigation in future studies.

The SNR of muscle was also observed to be unaltered between different time points for both T_2_ and T_2_* weighted imaging.

### Histological analysis

The time from USPIO infusion to endarterectomy ranged from 48 hrs to 70 days. Carotid plaques were retrieved from 6 patients scheduled for surgery and from these15 histological sections containing plaque available for analysis. All the sections were stained with H&E and EVG stains to demonstrate plaque morphology.

**Figure 6:**
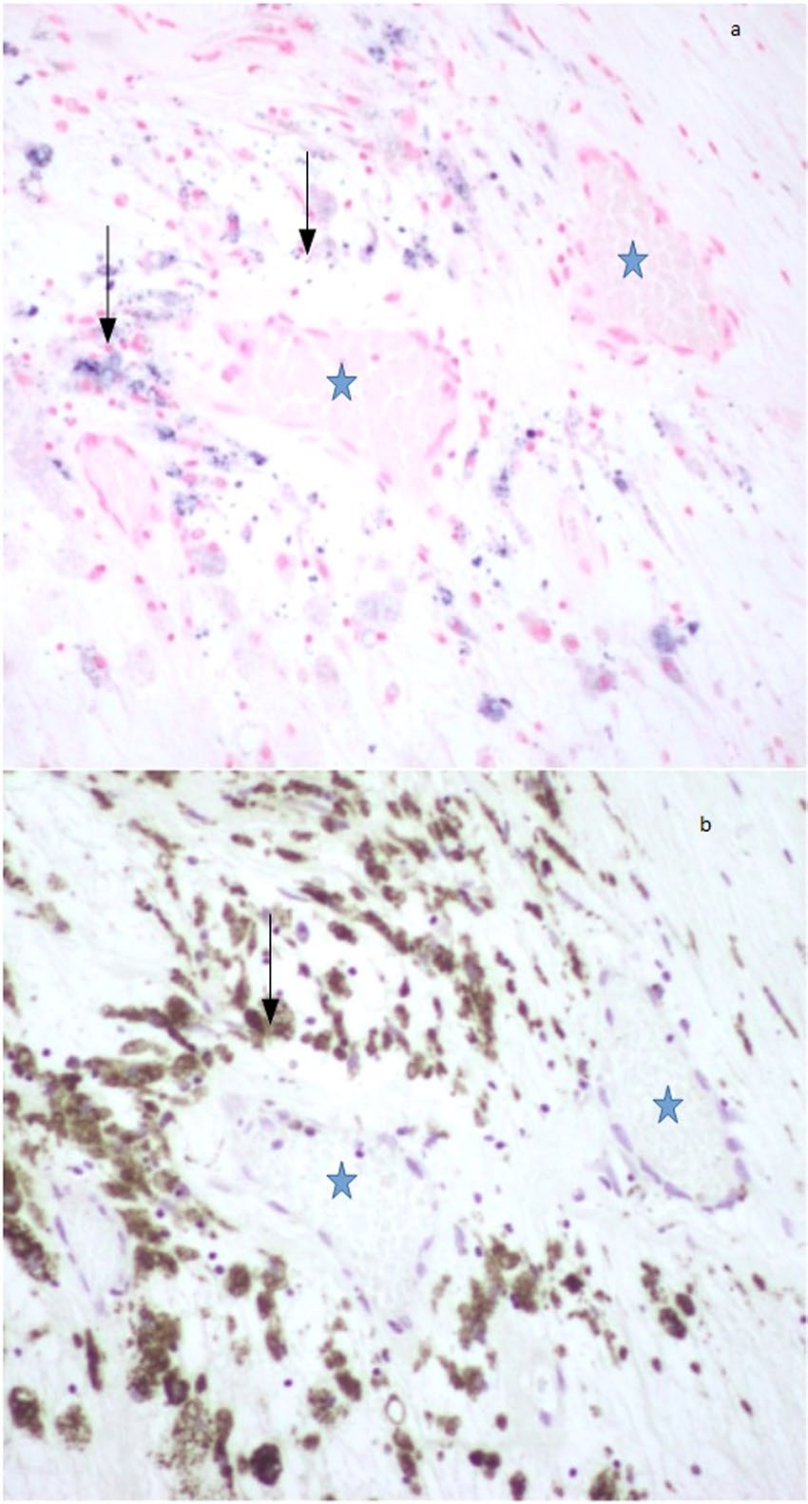
(**a**) Iron within ferumoxytol stained blue on Perl’s stain- back arrows (at 20 × magnification). (**b**) Immunohistochemical staining of macrophages (CD68) demonstrates the co-localization of the macrophages (black arrows) in the area corresponding to the ferumoxytol uptake on Perl’s stain (**a**). Neovessels (blue star) seem to be in vicinity of areas abundant in macrophages.

Perls-positive staining was observed in 13 sections from 6 of 6 patients demonstrating USPIO in multiple dissociated regions within the plaque, including the FC, LC, plaque shoulder and adventitia of the vessel wall. Macrophage immunostaining with CD68 antibody marker, co-localised with Perls-positive regions in all the 13 sections from the 6 patients was observed (see Fig. 6).

These results support our group previous histopathogical findings using Ferumoxtran-10^13^. Neovascularisation adjacent to the distribution of the macrophages was also observed hence indicative of the inflammation along with neovessel formation (see Fig. 6).

### Ferumoxytol-enhanced MR imaging of patients with symptomatic and asymptomatic carotid artery disease

All patients (n = 20) underwent ferumoxytol-enhanced MR imaging of carotid arteries successfully and tolerated the ferumoxytol infusion with no side effects. Patient demographics are presented in Table 5. The two clinical groups of symptomatic and asymptomatic patients had comparable demographics and co-morbidities (Tables 5 and 6). Total imaging time was ~25 minutes.

**Table 5:** Patient demographics.

Characteristics	n (%)
Number of patients	20
Male/ Female	17/3
Median age (years) (IQR)	71(66–75)
Mean luminal stenosis (North American Symptomatic Carotid Endarterectomy Trial) (%) [IQR]	58.7 (51.4–64.4)
Previous TIA (6 months ago) or asymptomatic	10 (50%)
Recent TIA/ nondisabling stroke	10 (50%)
Hyperlipidaemia	10 (50%)
Hypertension	17 (85%)
Diabetes Mellitus	5 (25%)
Peripheral arterial disease	2 (10%)
Previous coronary revascularisation	2(10%)
Ischaemic heart disease	5(25%)
Atrial fibrillation	0
Statin	20 (100%)
Clopidogrel	14(70%)
Aspirin	14 (70%)
Current or ex-smoker	13(65%)

**Table 6:** Comparison of the demographics and comorbidities of patients with symptomatic and asymptomatic carotid artery disease.

	Symptomatic carotid artery disease (n = 10)	Asymptomatic carotid artery disease (n = 10)	p-value (two-tailed)
Age (years)	70.5 (66.5–75.2)	70 (66–75.5)	0.548^†^
Vascular events (TIA/Stroke)	10 (100%)	4 (40%)	0.014*
Ischemic heart disease	2 (20%)	4 (40%)	0.62*
Carotid endarterectomy	4 (40%)	4 (40%)	1.00*
Hypertension	9 (90%)	8 (80%	0.53*
Hyperlipidaemia	8 (80%)	4 (40%)	0.53*
Diabetes	3 (30%)	2 (20%)	0.60*
Statin	10 (100%)	10 (100%)	1.00*
Clopidogrel	10 (100%)	4 (40%)	0.02*
Aspirin	9 (90%)	5 (50%)	0.14*
Angiotensin-converting enzyme inhibitor-Inhibitor	4 (40%)	3 (30%)	0.63*

*Chi-square, ^†^Mann-Whitney Test.

Signal loss was identified in all plaques on qT_2_ and qT_2_* sequences. Mean T_2_ and T_2_* values of the whole plaque on pre- and post-ferumoxytol imaging session were used for statistical analysis.

**Figure 7:**
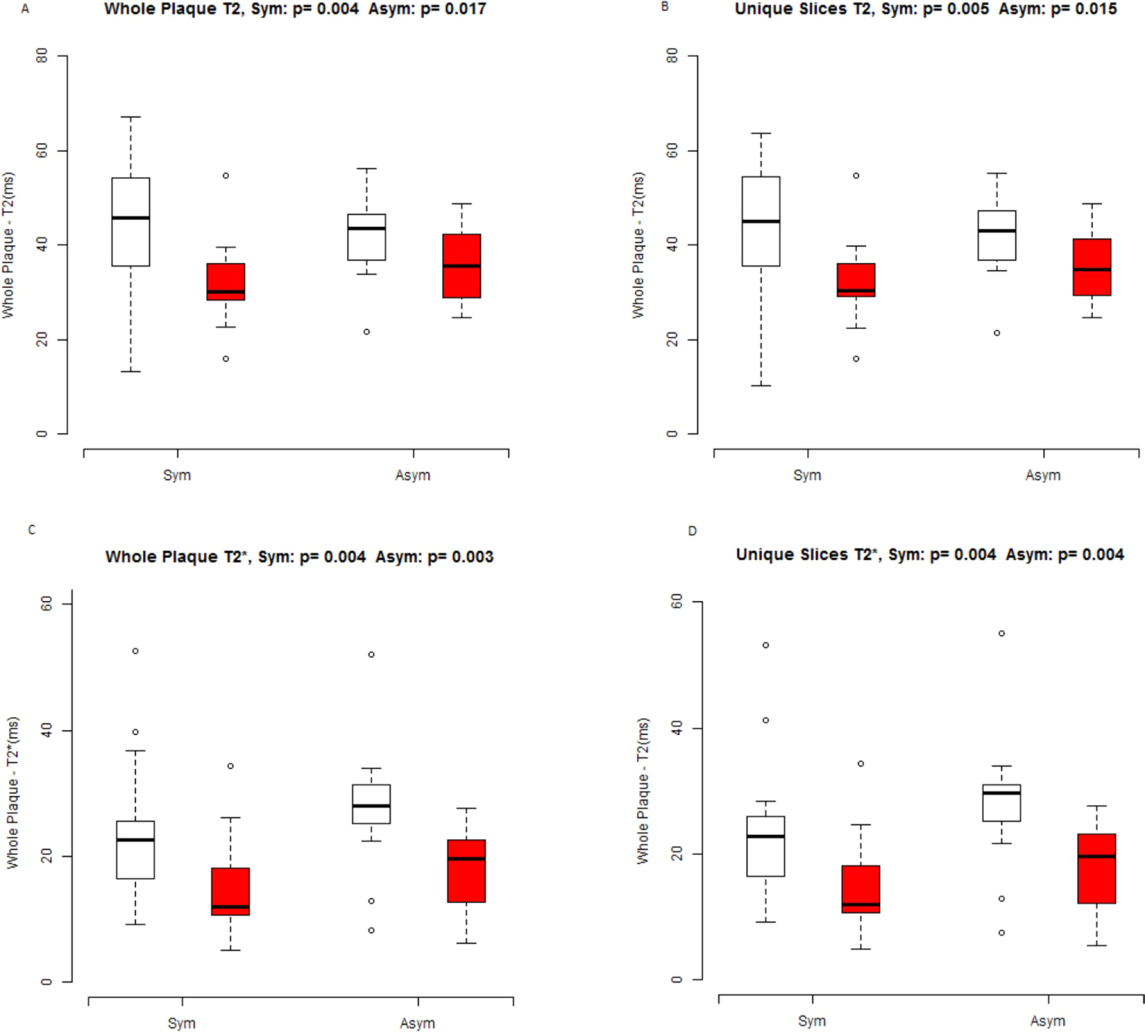
Box and whisker plots illustrates the Ferumoxytol uptake by symptomatic and asymptomatic patient cohort at baseline and 48 hrs post ferumoxytol MR imaging (**A**) the ∆ T 2 values, p = 0.030, p = 0.002 for symptomatic and asymptomatic patient cohort respectively. (**B**) The ∆T2*values, p = 0.003, p < 0.001 for symptomatic and asymptomatic cohort respectively.

**Figure 8:**
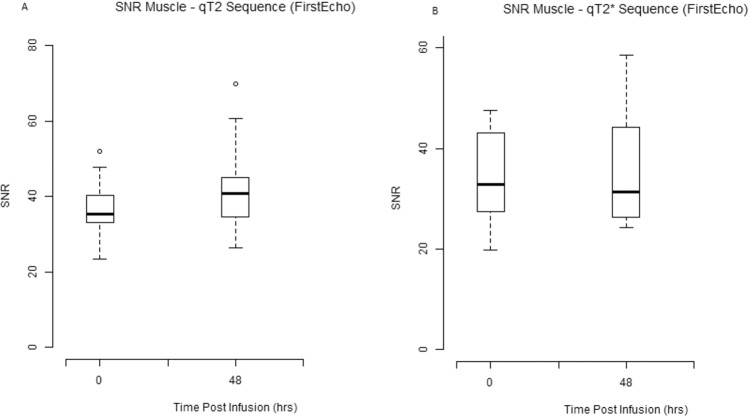
Box and whisker plots illustrate signal-to-noise ratio muscle on qT2* and qT2 weighted sequences at pre and 48hrs post ferumoxytol MR imaging (**A**) SNR muscle on qT2* weighted sequence (**B**) SNR muscle on qT2 weighted sequence at baseline and 48 hrs post-ferumoxytol MR imaging sessions.

**Figure 9:**
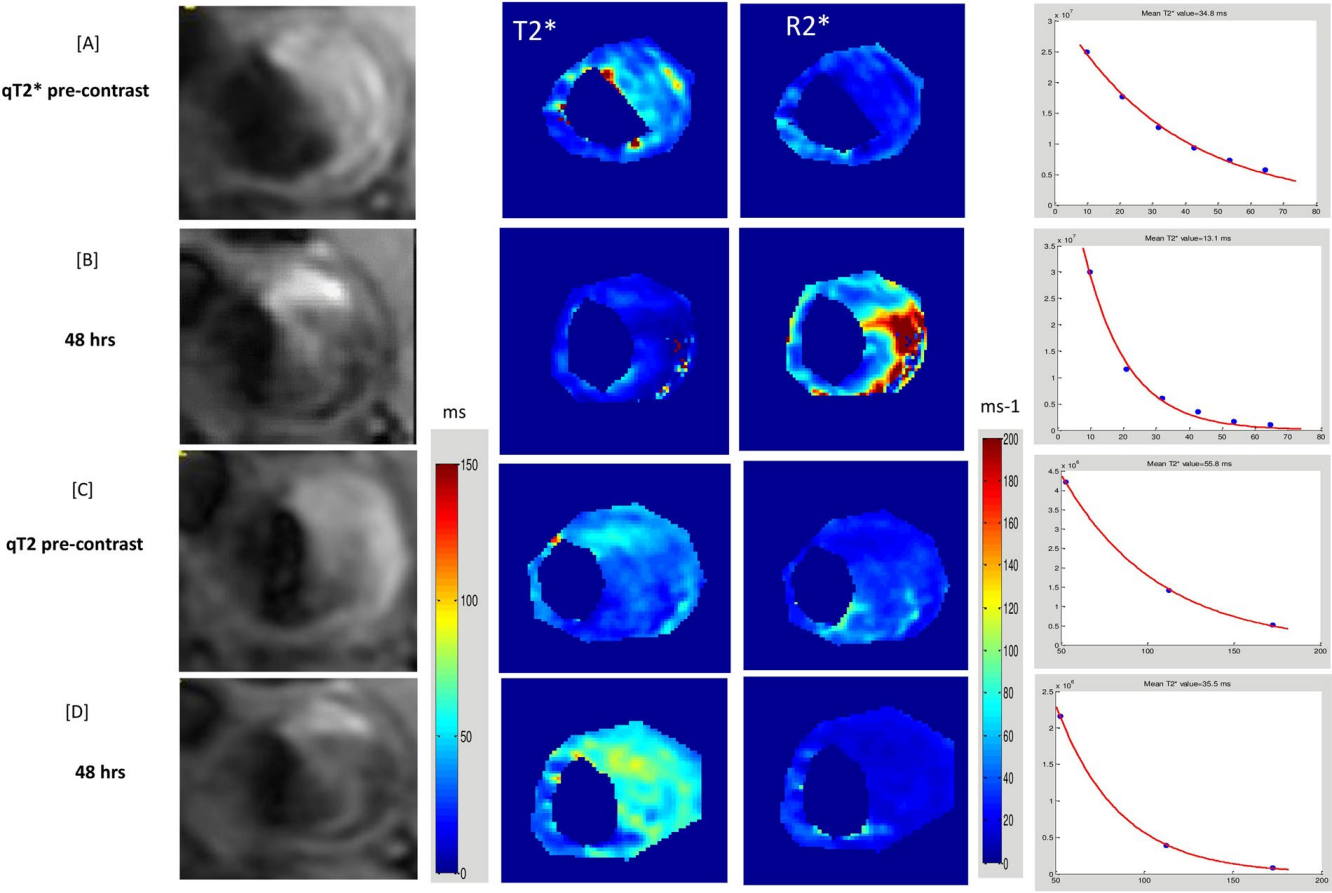
Representative quantitative T2* and quantitative T2 mapping images pre and 48hrs post ferumoxytol and corresponding R2*/T2*maps of carotid atheroma of a patient with asymptomatic carotid artery disease. (**A**) Pre-contrast qT2* = 34.8 ms (**B**) Post ferumoxytol qT2*with corresponding R2*/T2*maps and graphs demonstrating mean T2*values = 13.1 ms (**C**) Pre-contrast qT2 = 56.8 ms (**D**) Post ferumoxytol qT2 with corresponding R2*/T2*maps and graphs demonstrating mean T2*values of 35.5 ms.

In both symptomatic (n = 10) and asymptomatic (n = 10) patient cohorts, we observed a significant decrease in qT_2_ and qT_2_* values between baseline and 48 hrs post-ferumoxytol MR imaging session (∆ T_2_, p = 0.030; ∆T_2_*, p = 0.003) and (∆T_2_, p = 0.002; ∆T_2_*, p < 0.001) respectively (see Fig. 7). The SNR of muscle was observed to be unaltered between different time points for both T_2_ and T_2_* weighted imaging (see Fig. 8). T_2_*/R_2_* maps for an asymptomatic patient are presented in Fig. 9.

## Discussion

In this study we report the temporal dependence for ferumoxytol-enhanced MR imaging of human carotid atheroma for the first time in published literature. The feasibility of ferumoxytol in evaluation of carotid atheroma using blood suppressed qT_2_ and qT_2_* 3D pulse sequences in patients with symptomatic and asymptomatic carotid artery disease is also reported. Previously conducted studies in various vascular territories have used different imaging windows following the ferumoxytol administration^12,20^, however the optimum MR imaging window post-ferumoxytol remained widely unexplored. We found signal loss occurred between 24–72hrs with a significant statistical correlation between the qT_2_ and qT_2_* values at 48 hrs post ferumoxytol infusion. This was quantified using an advanced 3D acquisition technique and assessment of ΔqT_2_ and ΔqT_2_* values. The blood suppression technique, DANTE was utilised for T_2_* mapping which is superior to the previously used 2D acquisition methodologies. 3D preparation pulses achieve better blood suppression than 2D pulse sequences and allows 3D acquisition allows larger longitudinal coverage within feasible imaging times with respect to 2D techniques^10^. Besides, the T_2_* weighted multi-echo fast gradient echo pulse sequence requires high uniformity of the magnetic field, independent of the paramagnetic substance or diamagnetic substance therefore with changes in the uniformity of the magnetic field, the sequence is sensitive for detection of small lesions. Hence it has the ability to sensitively detect paramagnetic material, such as USPIO^21^.

This study reports the simultaneous acquisition of qT_2_ and qT_2_* 3D pulse sequences and qT_2_ and qT_2_* values in assessing the temporal dependency of ferumoxytol-enhanced MR imaging of carotid atheroma. To our knowledge this is the first report in the published literature to have used this comprehensive quantitative analysis for the evaluation of ferumoxytol-enhanced MR imaging in carotid artery territory. Development of this quantitative mapping method has been described by our group previously^15^.

Previous studies used the semi-quantitative analysis technique to assess the changes in relative signal intensities of the arterial wall normalized for the signal intensity in the adjacent muscle on T_2_ and T_2_* weighted images. However, this approach has been criticised as the differences in patient and coil positioning during subsequent imaging sessions may influence signal intensities in plaque area hence may lead to false interpretation of inflammation^22^. However, quantitative mapping techniques negate these confounding factors while enhancing the sensitivity of assessment of localisation and amount of USPIO uptake in atherosclerotic tissue. Using an advanced 3D acquisition technique, we were able to generate pixel-wise ∆T_2_/R_2_ and ∆T_2_*/R_2_* maps (R_2_* is the inverse of the mean T_2_*) from the pre and post contrast studies. A similar approach was recently used by another group for carotid imaging^12^. We have reported our results as decrease in qT_2_/qT_2_*, previously the comparison of the repeatability of R_2_* values and T_2_* values have been experimentally determined and no evidence of bias was observed^23^.

We were able to quantify signal loss in all patients who had ferumoxytol-enhanced MR imaging. This positive finding was best visualised using a blood-suppressed 3D T_2_*-weighted MEFGRE sequence between 48–72 hrs after infusion. We observed a decrease in T_2_/T_2_* values 24 hrs after USPIO administration. This was however associated with artefacts caused by insufficient blood suppression most likely due to the presence of ferumoxytol in the blood pool at this time-point. The decreased T_1_ of blood after ferumoxytol infusion would likely influence the accuracy of T_2_/T_2_* measurements at this time-point. The multi-echo fast gradient echo T_2_* weighted sequence is also sensitive to the smallest changes in magnetic field, hence when the paramagnetic contrast medium (like ferumoxytol) reaches the neovessel of the atherosclerotic plaque for the first time, the sequence can detect these changes sensitively, which appears as a decrease in signal intensity on this pulse sequence and simultaneous amplification of artefacts caused by insufficient blood suppression due to above reason^15^.

Using a quantitative approach, we observed a significant decrease in T_2_ and T_2_* values in carotid atheroma 48 hrs after ferumoxytol administration. As a quantitative indicator, the T_2_* value is widely used in clinical applications. Besides providing the measurements of the T_2_* value of tissues on multi-echo fast gradient echo T_2_* weighted imaging it also has the ability to indirectly reflect the iron content of the tissues. The increase in iron content of tissues can cause a shortening in the T_2_* relaxation time. This is conducive to quantitatively study iron uptake and deposition. This technique has been previously validated for the quantitative determination of changes in tissue biochemical components^24^. This decrease in T_2_* and T_2_ was not observed in the adjacent sternocleidomastoid muscle taken as a control. In this study we measured both T_2_ and T_2_* relativities and found them to be significantly correlated (r = 0.492) post-USPIO infusion at 48 hrs.

This fundamental information about the temporal dependence of ferumoxytol may potentially form the basis of use of this pharmaceutical agent in future studies aimed at assessing pathophysiology of atherosclerosis and atherosclerosis-related inflammation; and in determining efficacy of established and novel anti-atherosclerotic drugs using MR imaging studies.

We also observed that signal loss was still present at 96 hrs imaging in relation to the baseline in our study. This was evident on the decreased qT_2_ and qT_2_* values at 96 hrs imaging session. Previously conducted studies have shown the washout time for ferumoxytol in another vascular bed of approximately two weeks following the ferumoxytol administration^20^. As USPIOs are composed of an iron oxide core surrounded by a thick and complete dextran coat having a hydrodynamic size smaller than 50 nm. These particles remain mono-dispersed in solution and are withdrawn from the blood by MPS. The smaller size of these particles facilitates their extravasation through diseased microvessels, where they are engulfed and accumulated by the tissue-resident macrophages within the first 24 hours after the infusion^25^. Hence this macrophage-selective property allows imaging of vascular inflammation by delayed MR imaging.

Assessment of histological data revealed co-localisation of ferumoxytol and macrophages carotid atheroma. The presence of adjacent prominent neovessel alongside the ferumoxytol aggregation also supports the probable role of the nevessels in promoting macrophage recruitment because iron particles were found both within the adventitia and the plaque area.in areas. The exact mechanism of USPIOs uptake is however unknown.

In the second part of this exploratory study it was observed that:

1. In the symptomatic patient cohort, signal loss was observed as significant decrease in qT_2_ and qT_2_* values and signal loss on qT_2_ and qT_2_* weighted pulse sequences on post-ferumoxytol MR imaging with p = 0.030, p = 0.003 respectively. These findings support the initial hypothesis that patients with active disease would have higher macrophage activity and inflammation within atheroma and hence greater uptake of ferumoxytol by these activated macrophages resulting in significant signal drop on MR imaging.
2. It was observed that despite of the asymptomatic status of the study subjects, 90% of asymptomatic plaques demonstrated ferumoxytol uptake in the form of signal loss and decrease in qT_2_ and qT_2_* values on post-ferumoxytol MR imaging, p = 0.002 and p < 0.001 respectively. Since most of the patients in the asymptomatic patient cohort had moderate to severe stenosis, these findings may suggest that despite of their asymptomatic disease status for previous 6 months, there may be underlying inflammatory activity within atheroma. Also 40% of these patients had ischaemic heart disease, hence it may be inferred that despite of being asymptomatic in carotid territory, the inflammatory activity in the other vascular bed i.e. coronary bed may influence the level of inflammatory activity in carotid atheroma due to systemic inflammatory nature of atherosclerosis. Similar results were observed in previously conducted studies using ferumoxtran-10-enhanced MR imaging^6^.
3. On comparative quantitative analysis of both symptomatic and asymptomatic patient cohorts on qT_2_w and qT_2_*w imaging, no statistical significance was observed in this comparison (∆T_2_, p = 0.220; ∆T_2_*, p = 0.589 respectively). This is most likely a type 2 error, attributed to the relatively small sample size. Future studies are warranted to investigate this in an adequately powered study.

All patients recruited in this study tolerated the ferumoxytol infusion well with no significant adverse events and completed the consecutive imaging sessions. All the patients were closely monitored for any signs of hypersensitivity after the warning from The Food and Drug Administration on the use of ferumoxytol. We strictly adhered to the precautions as recommended in the guidelines by The Food and Drug Administration for the safe administration of this infusion. The safety data regarding the administration of ferumoxytol in our study further strengthen the potential use of USPIOs for vessel wall imaging. Given the availability of superparamagnetic iron oxide, ferumoxytol-enhanced MR imaging avoids the risk of nephrogenic systemic fibrosis, which has been associated with the administration of gadolinium-based contrast media, hence it could be a good alternative for vascular imaging as supported by the recently conducted trials^26,27^.

## Limitations

One of the potential issues relating to the uptake of ferumoxytol by macrophages is that the resulting susceptibility artefact may preclude short-term follow-up with other diagnostic MR imaging sequences; this issue may be of particular concern for follow-up hepatic or oncologic imaging because ferumoxytol can persist in macrophages for up to 2 months^28^. Also complete wash out of the previous dose of USPIO, allowing baseline T_2_* to return to normal is essential to reassess uptake of USPIO accurately, without impact from previous USPIO administration^20^.

### Other limitations include

(1) image quality affected by poor blood suppression as ferumoxytol remains intravascularly within first 24 hrs, hence imaging quality between 24–36 hrs is markedly affected till the time it is taken up intracellularly; (2) the decrease in T_1_ of blood following ferumoxytol administration may affect the accuracy of R_2_*measurements; (3) An additional patient visit posing a logistical limitation with MRI scheduling; (4) Inter-reader and Intrareader reproducibility was not assessed in this study. The lack of formal blinding of imaging prior to analysis was also a limitation. Future studies are warranted to assess this. (5) The second part of this study, being exploratory in nature, had small sample size. This was designed as a pilot study primarily to assess the feasibility of ferumoxytol-enhanced MR imaging in patients with symptomatic and asymptomatic disease. (6) There was variable duration between a patient undergoing carotid MR and carotid endarterectomy (maximum 70 days). This delay in obtaining the carotid sample for histological assessment may have affected some histology findings, however we consistently noted co-localisation of Perl’s stained iron with areas containing CD68 stained macrophages. (8) There were only 2 female patients in our study sample. It remains predominantly an unanswered question about how the immune mechanistics differs between men and women regarding atherosclerosis^29^, and its implications for imaging studies using iron oxide contrast media for investigating atherosclerosis related inflammation.

## Conclusions

This study demonstrates the feasibility and temporal dependence of ferumoxytol-enhanced MR imaging of carotid atheroma. This was visualised as foci of signal reduction that are attributed to ferumoxytol uptake by macrophages which was evident on histopathological assessment. The optimum imaging window with maximum signal drop during serial MR imaging sessions was identified to be 48 hrs post ferumoxytol infusion. The precise quantifiable indicators such as T_2_* relaxation time may prove to be more conducive for the quantitative evaluation of the physiological/pathological status of the tissues. These preliminary results are encouraging and offer a rationale to conduct further investigation on larger patient cohorts.

The fundamental information about the temporal dependence of ferumoxytol may potentially form the basis of use of this pharmaceutical agent in future studies aimed at assessing pathophysiology of atherosclerosis and atherosclerosis-related inflammation; and in determining efficacy of established and novel anti-atherosclerotic drugs using MR imaging studies.

## Data Availability

Authors agree to making materials, data and associated protocols promptly available to readers without undue qualifications in material transfer agreements as required.

## References

[1] Libby P, Lichtman AH, Hansson GK (2013). Immune effector mechanisms implicated in atherosclerosis: from mice to humans. Immun..

[2] Moreno PR, Purushothaman M, Purushothaman KR (2012). Plaque neovascularization: defense mechanisms, betrayal, or a war in progress. Ann. N. Y. Acad. Sci..

[3] Sadat U (2014). Inflammation and neovascularization intertwined in atherosclerosis: imaging of structural and molecular imaging targets. Circulation.

[4] Chinetti-Gbaguidi G, Colin S, Staels B (2015). Macrophage subsets in atherosclerosis. Nat. Rev. Cardiol..

[5] Falk E, Nakano M, Bentzon JF, Finn AV, Virmani R (2013). Update on acute coronary syndromes: the pathologists’ view. Eur. Heart J..

[6] Tang TY (2008). Comparison of the inflammatory burden of truly asymptomatic carotid atheroma with atherosclerotic plaques in patients with asymptomatic carotid stenosis undergoing coronary artery bypass grafting: an ultrasmall superparamagnetic iron oxide enhanced magnetic resonance study. Eur. J. Vasc. Endovasc. Surg..

[7] Trivedi RA, U-King-Im JM, Graves MJ, Kirkpatrick PJ, Gillard JH (2004). Noninvasive imaging of carotid plaque inflammation. Neurol..

[8] Tang TY (2009). The ATHEROMA (Atorvastatin Therapy: Effects on Reduction of Macrophage Activity) Study. Evaluation using ultrasmall superparamagnetic iron oxide-enhanced magnetic resonance imaging in carotid disease. J. Am. Coll. Cardiol..

[9] Trivedi RA (2004). *In vivo* detection of macrophages in human carotid atheroma: temporal dependence of ultrasmall superparamagnetic particles of iron oxide-enhanced MRI. Stroke.

[10] Tang TY (2009). Temporal dependence of *in vivo* USPIO-enhanced MRI signal changes in human carotid atheromatous plaques. Neuroradiology.

[11] Weinstein JS (2010). Superparamagnetic iron oxide nanoparticles: diagnostic magnetic resonance imaging and potential therapeutic applications in neurooncology and central nervous system inflammatory pathologies, a review. J. Cereb. Blood Flow. Metab..

[12] Smits LP (2017). Evaluation of ultrasmall superparamagnetic iron-oxide (USPIO) enhanced MRI with ferumoxytol to quantify arterial wall inflammation. Atherosclerosis.

[13] McBride OM (2016). Positron Emission Tomography and Magnetic Resonance Imaging of Cellular Inflammation in Patients with Abdominal Aortic Aneurysms. Eur. J. Vasc. Endovasc. Surg..

[14] Investigators MRS (2017). Aortic Wall Inflammation Predicts Abdominal Aortic Aneurysm Expansion, Rupture, and Need for Surgical Repair. Circulation.

[15] Yuan J (2017). The development and optimisation of 3D black-blood R2* mapping of the carotid artery wall. Magn. Reson. Imaging.

[16] Yuan J (2019). A Comparison of Black-blood T2 Mapping Sequences for Carotid Vessel Wall Imaging at 3T: An Assessment of Accuracy and Repeatability. Magn. Reson. Med. Sci..

[17] Spinowitz BS (2008). Ferumoxytol for treating iron deficiency anemia in CKD. J. Am. Soc. Nephrol..

[18] Dosa E (2011). MRI using ferumoxytol improves the visualization of central nervous system vascular malformations. Stroke.

[19] Constantinides CD, Atalar E, McVeigh ER (1997). Signal-to-noise measurements in magnitude images from NMR phased arrays. Magn. Reson. Med..

[20] Stirrat CG (2017). Ferumoxytol-enhanced magnetic resonance imaging assessing inflammation after myocardial infarction. Heart.

[21] Yang YM (2014). Comparison of USPIO-enhanced MRI and Gd-DTPA enhancement during the subacute stage of focal cerebral ischemia in rats. Acta Radiol..

[22] Fayad ZA, Razzouk L, Briley-Saebo KC, Mani V (2009). Iron oxide magnetic resonance imaging for atherosclerosis therapeutic evaluation: still “rusty?”. J. Am. Coll. Cardiol..

[23] Alam SR (2012). Ultrasmall superparamagnetic particles of iron oxide in patients with acute myocardial infarction: early clinical experience. Circ. Cardiovasc. Imaging.

[24] Lin ZC, Zhai L, Chne YP, Zhang XL (2011). Clinical application of T2*GRE multiple echo sequence on articular cartilage disease in the knee. Nan Fang. Yi Ke Da Xue Xue Bao.

[25] Hasan DM (2012). Macrophage imaging within human cerebral aneurysms wall using ferumoxytol-enhanced MRI: a pilot study. Arterioscler. Thromb. Vasc. Biol..

[26] Neuwelt EA (2009). Ultrasmall superparamagnetic iron oxides (USPIOs): a future alternative magnetic resonance (MR) contrast agent for patients at risk for nephrogenic systemic fibrosis (NSF)?. Kidney Int..

[27] Mukundan S (2016). Ferumoxytol-Enhanced Magnetic Resonance Imaging in Late-Stage CKD. Am. J. Kidney Dis..

[28] Ittrich H, Peldschus K, Raabe N, Kaul M, Adam G (2013). Superparamagnetic iron oxide nanoparticles in biomedicine: applications and developments in diagnostics and therapy. Rofo.

[29] Fairweather D (2014). Sex differences in inflammation during atherosclerosis. Clin. Med. Insights Cardiol..

